# A novel two-step, direct-to-PCR method for virus detection off swabs using human coronavirus 229E

**DOI:** 10.1186/s12985-020-01405-y

**Published:** 2020-08-25

**Authors:** Zachary P. Morehouse, Caleb M. Proctor, Gabriella L. Ryan, Rodney J. Nash

**Affiliations:** 1 Michigan State University College of Osteopathic Medicine, East Lansing, MI USA; 2 Omni International Inc, Kennesaw, GA USA; 3 Department of Biology, Georgia State University, 100 Peidmont Ave SE, 4th Floor, Atlanta, GA 30303 USA; 4 Jeevan Biosciences, Tucker, GA USA

**Keywords:** Virus detection, Viral diagnostics, PCR, Coronavirus

## Abstract

**Background:**

Currently, one of the most reliable methods for viral infection detection are polymerase chain reaction (PCR) based assays. This process is time and resource heavy, requiring multiple steps of lysis, extraction, purification, and amplification procedures. Herein, we have developed a method to detect virus off swabs using solely shaker-mill based mechanical lysis and the transfer of the viral lysate directly to a PCR assay for virus detection, bypassing the substantial reagent and time investments required for extraction and purification steps.

**Methods:**

Using Human Coronavirus 229E (HCoV-229E) as a model system, we spiked swabs in vitro for proof-of-concept testing. Swabs were spiked in serial dilutions from 1.2 × 10^6^ to 1.2 × 10^1^ copies/mL and then placed in 2 mL tubes with viral transport media (VTM) to mimic the specimen collection procedures in the clinic prior to processing via shaker-mill homogenization. After homogenization, 1 μL of lysate was processed using RT-qPCR for amplification of the nucleocapsid (N) gene, qualifying viral detection.

**Results:**

HCoV-229E in vitro spiked swabs were processed in a novel two-step, direct-to-PCR methodology for viral detection. After running 54 swabs, we confidently determined our limit of detection to be 1.2 × 10^3^ viral copies/mL with 96.30% sensitivity.

**Conclusion:**

We have proven that the shaker-mill homogenization-based two-step, direct-to-PCR procedures provides sufficient viral lysis off swabs, where the resulting lysate can be used directly in PCR for the detection of HCoV-229E. This finding allows for reductions in the time and resources required for PCR based virus detection in comparison to the traditional extraction-to-PCR methodology.

## Introduction

As the number of viral diseases are on the rise, it is critical to continue to innovate and advance diagnostic, treatment, and surveillance methods surrounding viral infections. Herein, we are proposing a novel method for viral pathogen detection off swabs as an improvement or alternative to the current PCR based assays commonly used for viral detection [1, 2]. The traditional protocol for preparing a sample for PCR based detection often involves procedures of swabbing a patient, processing the sample to lyse the virus, extract, and purify its nucleotides, and then amplify the purified genetic material via PCR for detection of a gene product needed to confirm the patient’s suspected diagnosis [3]. In the face of the COVID-19 pandemic, attempts have been made to perform PCR based diagnostics for SARS-CoV-2 with reduced time and cost, especially as the plastics and reagents needed for traditional viral nucleotide extractions have become scarce in the face of an exponentially increased global demand [4–7]. Though some success has been seen with thermal and enzymatic digestion attempts on viral samples to expose the genetic material for extraction-less PCR detection, no method has shown high viral lysis in under 1 min when dealing with clinical concentrations of virus on swabs [5, 6, 8].

Shaker-mill homogenization as a form of mechanical lysis has been proven time and again as a successful method for disrupting tissues, microorganisms, and biologic samples for downstream molecular analysis; however, these downstream applications often require the use of additional purification, isolation, or extraction procedures before those analyses can be completed [9–12]. While, shaker-mill homogenization procedures have not yet been applied directly to diagnostic technologies, its ability to lyse organisms and tissues far tougher than a virus have been well categorized [9–12]. Building off the increased need for novel viral diagnostic methodologies, which reduce the resources and time required for accurate viral pathogen detection, we felt it was time to examine the capabilities of shaker-mill homogenization for viral lysis to be used for downstream detection.

Using human coronavirus 229E (HCoV-229E) as our model organism, we developed a novel two-step methodology of optimized shaker-mill homogenization parameters that allowed for direct-to-PCR viral detection. HCoV-229E is an enveloped, positive-sense, single-stranded RNA virus [13]. As a known human pathogen, it HCoV-229E is one of the four most common circulating coronaviruses associated with mild to moderate respiratory illness globally [13]. It is commonly included in commercial respiratory viral panel screening as a source of the common cold [13]. HCoV-229E was the chosen model for this project as a biosafety level 2 pathogen with similar genetic and protein composition to the current SARS-CoV-2 virus for developing novel diagnostic approaches, until the process is proven in vitro [13, 14]. The linear genome and enveloped structure of HCoV-229E allows for this virus to be a sufficient in vitro substitute for SARS-CoV-2 during the early stages of methodology development described within this manuscript [13].

## Materials and methods

### Cell culture and virus growth

Human coronavirus 229E (HcoV-229E) (ATCC, Cat. No. VR-740) was added at a multiplicity of infection (MOI) of 1.0 to an approximately 85% confluent T75 flask of MRC-5 cells (ATCC, Cat. No. CCL-171), 48 h after plating. The flask was maintained with DMEM (Fisher Scientific, Cat. No. 11–965-118) supplemented with 5% heat inactivated fetal bovine serum (Gemini Bioproducts, Cat. No. 100–500) and 1% L-Glutamine (Gemini Bioproducts, Cat. No. 400–106), incubated at 37 °C with 5% CO_2_ [15]. The cell culture supernatant was harvested at 72 h post infection when 80% cytopathic effect (CPE) was observed.

### Viral transport media formulation and production

Viral transport media (VTM) was produced following the US CDC (Atlanta, GA, USA) guidelines, found freely available on their website for formulation of VTM in a laboratory as an alternative to commercial VTM purchases. 500 mL of Hanks Balanced Salt Solution (HBSS) 1X with calcium and magnesium ions (no phenol red) (Fisher Science, Cat. No. SH3058801) was supplemented with 2% heat inactivated fetal bovine serum (Gemini Bioproducts, Cat. No. 100–500). 100 mg of Gentamicin (Gemini Bioproducts, Cat. No. 400-100P) and 500 μg of Amphotericin B (Gemini Bioproducts, Cat. No. 400-104P) was added to the mixture and mixed thoroughly to create a final product of VIRAL TRANSPOT MEDIA, 2% FBS, 100 μg/mL Gentamicin, 0.5 μg/mL Amphotericin B. This viral transport media was used for storage and processing of all swab samples in this manuscript.

### Swab viral spike

Sterile cotton swabs (Fisher Science, Cat. No. 22–029-488) were submerged for 5 s in viral solutions ranging from 1.2 × 10^7^ to 1.2 × 10^1^ viral copies/mL [16]. The swabs were exposed in a serial dilution pattern, with three swabs being exposed at each concentration log to evaluate the detection capabilities of this method. The saturated swabs were then placed in a 2 mL screw capped tube (Omni International, Cat. No. 19–648) prefilled with 1 mL of viral transfer buffer [17]. The stem of the swab was then broken off at a level even with the top of the tube to allow for the cap to be screwed on for transporting and processing. The samples were prepared at 23 °C and then incubated for 1 h at 23 °C prior to processing.

### Shaker-mill swab processing for viral lysis

**Fig. 1:**
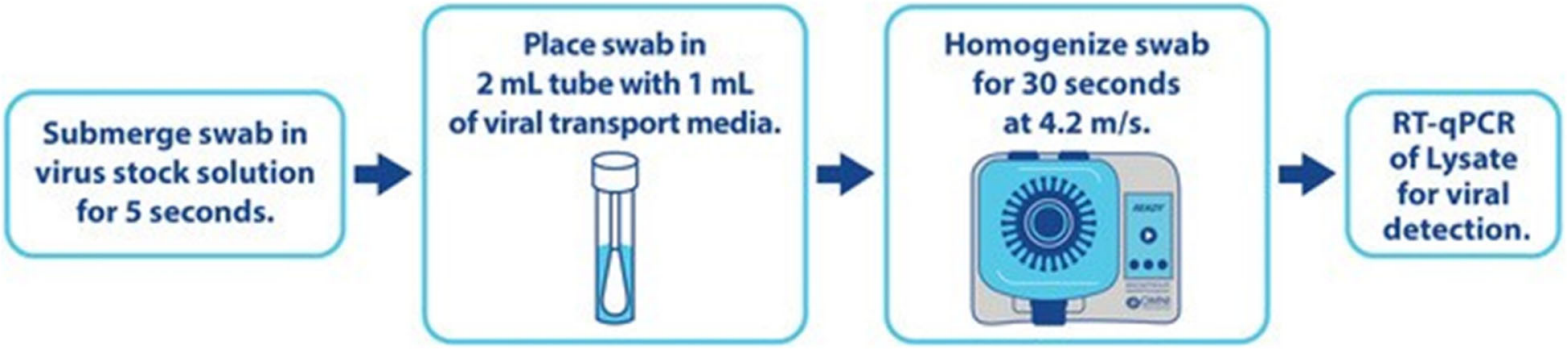
Graphical depiction of general two-step, direct-to-PCR in vitro testing method that was employed in all experiments discussed in this manuscript

To maintain optimal levels of biosafety, the following shaker-mill processing was completed in a biosafety cabinet to protect the user from any potential aerosol production during processing. Twenty-four 2 mL screw cap tubes containing the virally spiked swabs were processed on the Omni Bead Ruptor Elite (Omni International, Cat. No. 19-040E) for 30 s at 4.2 m/s. This processing generated froth within the tube which was allowed to settle prior to removal of 1 μL of lysate for RT-qPCR (Fig. 1).

### HcoV-229E RT-qPCR

HcoV-229E nucleocapsid gene (N gene) was selected as a target for RT-PCR from Vabret et al. [1, 18]. The N gene was targeted with forward primer 5′-AGGCGCAAGAATTCAGAACCAGAG-3′ and reverse primer 5′-AGCAGGACTCTGATTACGAGAAAG-3′ [1]. 1 μL of sample lysate was added to create a final reaction volume of 20 μL using the proportions of primers, sample, SYBR, RT, and DEPC-treated H_2_O as laid out in the New England Biologics Luna RT-qPCR Kit (NEB, Cat. No. E3005S). Amplification of lysate was performed for 44 cycles and the resulting amplicons were loaded into a 2% agarose (Bio-Rad, Cat. No. 1613101) gel for product visualization. Out of abundance of caution, the loading of the PCR plate with viral lysate should be completed in a biosafety cabinet to protect the user from any potentially viable virus particles remaining following shaker-mill homogenization.

### Plaque assay protocol for viral quantification

HcoV-229E was quantified with standard plaque assay protocols [19]. HEK-293 cells (ATCC, Cat. No. CRL-1573) were seeded at 2.0 × 10^5^ cells per well in 6 well tissue culture treated plates (Fisher Science, Cat. No. 07–200-601) with 2 mL of DMEM (Fisher Scientific, Cat. No. 11–965-118) infused with 5% heat inactivated fetal bovine serum (Gemini Bioproducts, Cat. No. 100–500). Once the cells achieved 85% confluence, 200 μL of HcoV-229E stock was added to the media of the first well. The media was gently mixed and 200 μL of the infected media was transferred into the adjacent well. This was repeated to create serial dilutions throughout the plate. After 24 h of incubation, the virally infected media was removed from each well and replaced with 2 mL of DMEM infused with 5% heat inactivated FBS and 2% agarose (Bio-Rad, Cat. No. 1613101). The plate was incubated for an additional 5 days at 35 °C with 5% CO_2_ and plaques were counted to determine viral concentration in plaque forming units/mL (PFU/mL).

## Results

**Fig. 2:**
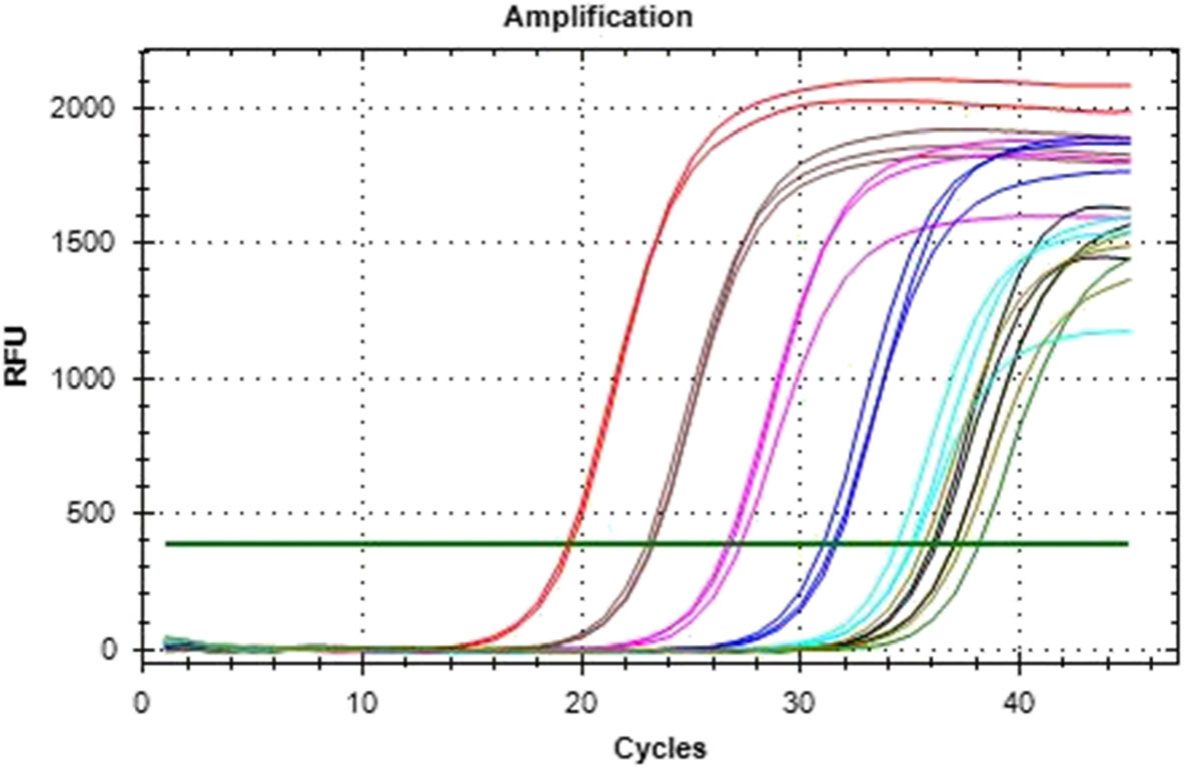
Two-step, direct-to-PCR results from serial dilution of viral concentration spiked onto swabs for limit of detection testing. Red lines represent 1.2 × 10^7^ viral copies/mL spiked swabs. Brown lines represent 1.2 × 10^6^ viral copies/mL spiked swabs. Pink lines represent 1.2 × 10^5^ viral copies/mL spiked swabs. Navy lines represent 1.2 × 10^4^ viral copies/mL spiked swabs. Teal lines represent 1.2 × 10^3^ viral copies/mL spiked swabs. Olive lines represent 1.2 × 10^2^ viral copies/mL spiked swabs. Black lines represent 1.2 × 10^1^ viral copies/mL spiked swabs. Green lines represent virus free, negative control swabs

**Fig. 3:**
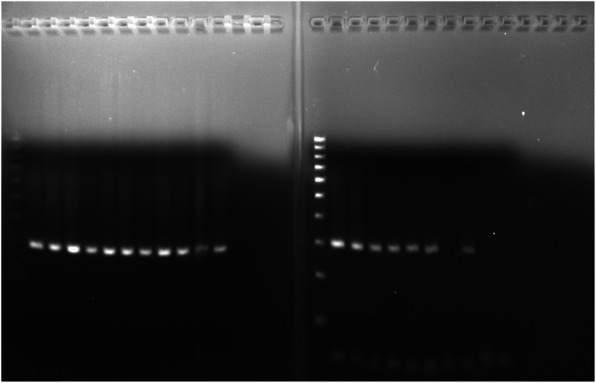
Gel visualization of amplicons from RT-qPCR shown in Fig. 2. Starting at the furthest left lane on the first gel, lane 1 is 100 bp DNA ladder, lanes 2 and 3 are amplicon visualization of 1.2 × 10^7^ viral copies/mL swab spike, lanes 4,5, and 6 are amplicon visualization of 1.2 × 10^6^ viral copies/mL swab spike, lanes 7,8, and 9 are amplicon visualization of 1.2 × 10^5^ viral copies/mL swab spike, lanes 10,11, and 12 are amplicon visualization of 1.2 × 10^4^ viral copies/mL swab spike, lanes 13,14,15 are empty. Starting with the furthest left lane on the second gel, lane 1 is 100 bp DNA ladder, lanes 2, 3, and 4 are amplicon visualization of 1.2 × 10^3^ viral copies/mL swab spike, lanes 5,6, and 7 are amplicon visualization of 1.2 × 10^2^ viral copies/mL swab spike, lanes 8, 9, and 10 are amplicon visualization of 1.2 × 10^1^ viral copies/mL swab spike, lanes 11,12, 13, 14, and 15 are empty. Visualization of a 308 bp amplicon is confirmatory of the HCoV-229E N gene

**Fig. 4:**
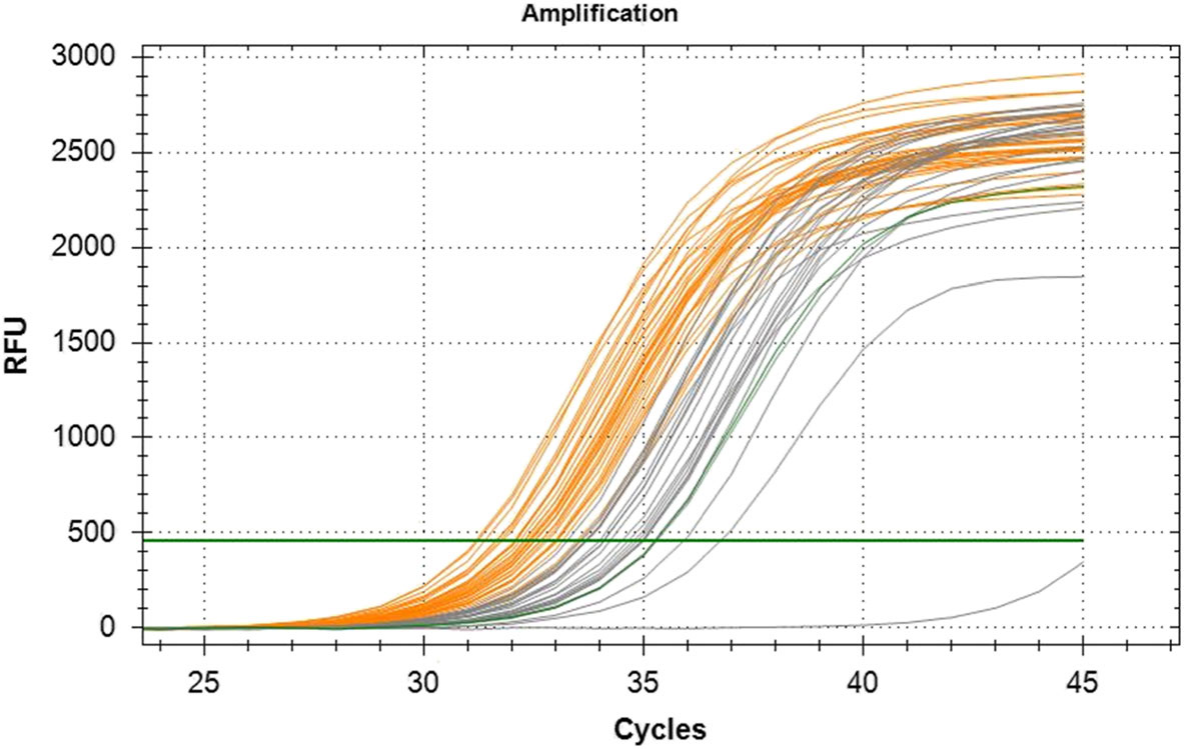
HCoV-229E N transcript detection using RT-qPCR following shaker-mill homogenization off swabs spiked in vitro with 1.2 × 10^3^ viral copies/mL. Orange lines represent viral stock 1 spiked swabs at 1.2 × 10^2^ viral copies/mL. Grey lines represent viral stock 2 spiked swabs at 1.2 × 10^2^ viral copies/mL. The green line represents a negative control, virus-free swab

**Fig. 5:**
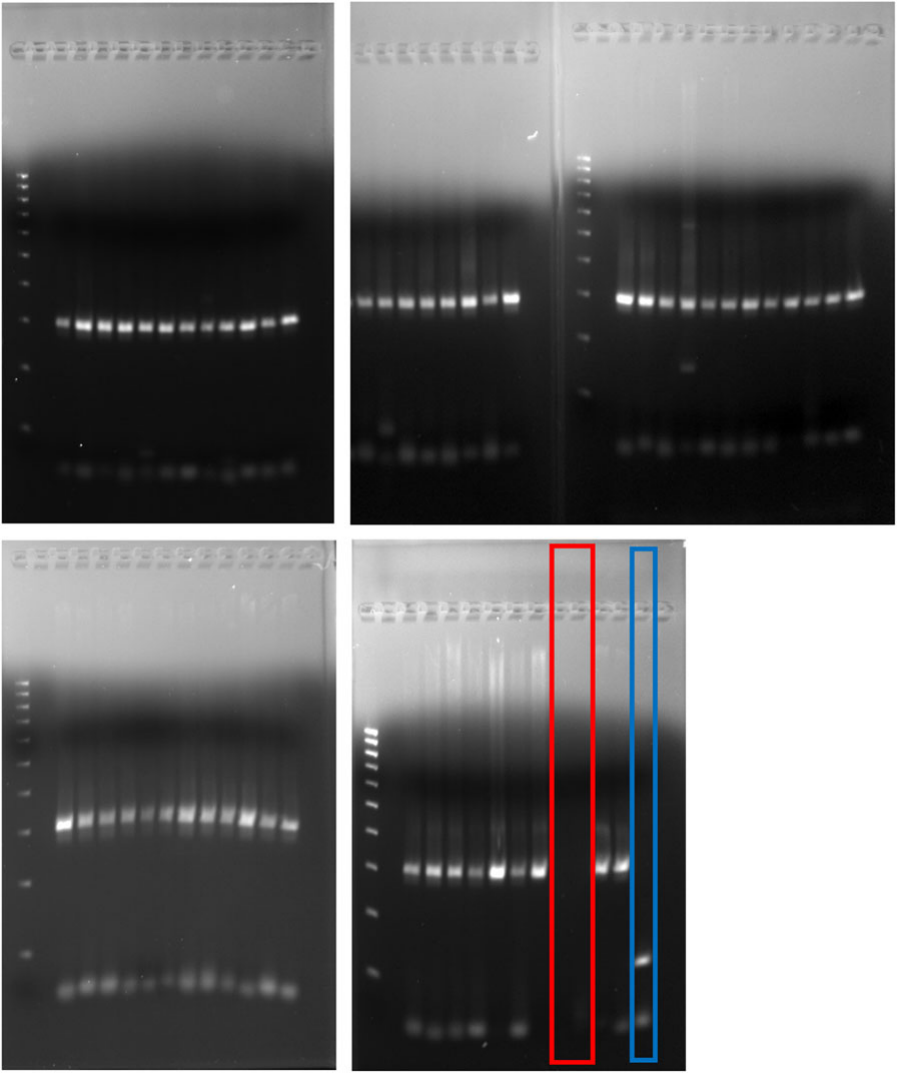
Gel visualization of the amplicons resulting from RT-qPCR done for method sensitivity testing at the lower limit of detection shown in Fig. 4. Visualization of a 308 bp amplicon is confirmatory of the HCoV-229E N gene. Note, of all 56 samples shown, only two swab samples (red box) and the one negative control (blue box) seen on the final gel of the series did not validate with amplicon visualization. All other unlabeled lanes are viral lysate produced N gene amplicon

We have successfully demonstrated that shaker-mill homogenization using the Omni Bead Ruptor Elite provides sufficient viral lysis from spiked swabs to allow for viral detection via direct RT-qPCR of the lysate. Using this novel method (Fig. 1) at our optimized run parameters of 4.2 m/s for 30s, we found the lower limit of reliable detection to be 1.2 × 10^3^ viral copies/mL (Figs. 2, 3). This limit of detection was evaluated for reproducibility by testing 56 in vitro spiked swabs from two different viral stock solutions, using RT-qPCR (Fig. 4). The results were confirmed via amplicon visualization (Fig. 5) and demonstrated a sensitivity of 96.30% when processing with this two-step, direct-to-PCR method.

In addition to the strong method sensitivity shown with in vitro testing, we also consistently showed greater than 90% viral lysis after shaker-mill homogenization for 30s at 4.2 m/s. Plaque assays were used to determine percent lysis following homogenization as shown with decreased PFU counts between stock and lysate (Table 1).

**Table 1:** Viral lysis data at different run parameters of shaker-mill homogenization as determined by plaque assay

Run Parameter	Average Lysis
4.2 m/s for 15 s	62.5%
4.2 m/s for 30s	97.0%
4.2 m/s for 60s	87.5%

## Discussion

The cost, time, and availability of current PCR assays have been considered prohibitive measures in the timely diagnosis and subsequent treatment of viral infections [20]. There is a critical need for the development of diagnostic technologies that help to improve access and reduce cost—this is especially important for increasing the treatment of infectious diseases in socioeconomically disenfranchised populations. While still in its infancy, it is our hope that this novel methodology can be applied as one potential solution for this need. The authors acknowledge that there are crucial next steps for clinical validation of this methodology but feel that the presented data is compelling and provides a strong starting point for this to occur. While these studies was completed using HcoV-229E as an in vitro model for methodology development geared towards SARS-CoV-2 applications, it is also our hope that this methodology can be further evaluated at both the benchtop and the bedside to be applied to other respiratory viruses also commonly tested for via nasopharyngeal or oropharyngeal swabs and PCR based detection.

In addition to the improvements to access of care, this two-step, direct-to-PCR methodology also has the potential to improve laboratory safety. As shown in Table 1, shaker-mill homogenization results in greater than 90% lysis of the virus in a sample. This high percentage of viral lysis demonstrates that there would be few, if any, viable viral particles left in the sample tube when the laboratory technician opens it for transfer to the RT-qPCR reaction. By using our two-step, direct-to-PCR methodology, the lab technician is also able to significantly reduce exposure to viral particles that would normally be increased when opening and closing sample tubes and adding buffers during traditional nucleic acid extraction processes. The low levels of viable and infectious viral particles when the sample is opened result in decreased exposure potential through laboratory accidents or aerosolization during sample opening that could result in laboratory acquired infections.

In contrast to the many benefits provided by this novel methodology, the authors do acknowledge that introducing a new approach to existing methods is difficult. We expect to see resistance from laboratories with currently validated testing methods, researchers with years of experience using the traditional extraction-to-PCR process, and other commercial entities involved in the currently accepted processes for PCR based viral diagnostics. However, it is our opinion that when advancements in technology and methodology can be made that will improve access to care, reduce cost, and improve safety, it is our responsibility to the public to explore these options. In the face of increasing virally caused infections globally, it is our duty to do everything we can to shake every tree and turn over every stone to prepare society to combat these diseases.

## Conclusion

We have successfully proven that shaker-mill homogenization provides sufficient viral lysis off swabs, where the resulting lysate can be used directly in PCR based assays for the detection of virus. This novel two-step, direct-to-PCR method for viral detection off swabs has shown a lower limit of reliable detection at 1.2 × 10^3^ viral copies/mL with 96.30% sensitivity in vitro when screening for HcoV-229E. Herein, we have demonstrated the success of this methodology in vitro and propose it as a novel approach to viral detection that allows for decrease run time in comparison to traditional PCR based viral detection assay protocols, as well as a reduction in the materials needed for successful viral detection. Bypassing standard extraction methods, while maintaining a lower limit of detection one log below the reported viral load on clinically obtained swabs positive for coronavirus [16], we demonstrated that this novel method has warranted further clinical evaluation for its potential to reduce the cost and time needed for each test.

## Data Availability

All data supporting this manuscript can be accessed by reaching out to the corresponding authors RJ Nash and ZP Morehouse directly at rnash2@gsu.edu and moreho17@msu.edu.

## Abbreviations

HCoV-229E: Human coronavirus 229E
PCR: Polymerase chain reaction
RT-qPCR: Quantitative reverse transcription polymerase chain reaction
RT: Reverse transcriptase
DMEM: Dibco Modified Eagles Media
VTM: Viral transport media
PFU: Plaque forming units

## References

[1] Vabret A, Mouthon F, Mourez T, Gouarin S, Petitijean J, Freymuth F (2001). Direct diagnosis of human respiratory coronaviruses 229E and OC43 by the polymerase chain reaction. J Virol Methods.

[2] Matoba Y, Abiko C, Ikeda T, Aoki Y, Suzuki Y, Yahagi K, Matsuzaki Y, Itagaki T, Katsushima F, Katsushima Y, Mizuta K (2014). Detection of the human coronavirus 229E, HKU1, NL63, and OC43 between 2010 and 2013 in Yamagata, Japan. Jpn J Infect Dis.

[3] Bustin SA, Nolan T (2020). RT-qPCR testing of SARS-CoV-2: a primer. Int J Mol Sci.

[4] Basu A, Zinger T, Inglima K, Woo KM, Atie O, Yurasits L, See B, Aguero-Rosenfeld ME. Perforrmance of Abbott ID NOW COVID-19 rapid nucleic acid amplification test in nasopharyngeal swabs transported in viral media and dry nasal swabs, in a New York City academic institution. J Clin Microbiol. 2020. 10.1128/JCM.01136-20 Online ahead of print. 10.1128/JCM.01136-20 PMC7383552 32471894

[5] Lu R, Wu X, Wan Z, Li Y, Jin X, Zhang C. A novel reverse transcription loop-mediated isothermal amplification method for rapid detection of SARS-CoV-2. 2020;21(8):2826. 10.3390/ijms21082826. 10.3390/ijms21082826 PMC7216271 32325642

[6] Fomsgaard AS, Rosenstierne MW (2020). An alternative workflow for molecular detection fo SARS-CoV-2 – Escape from the NA extraction kit-shortage, Copenhagen, Denmark, March 2020. Euro Surveill.

[7] Bosworth A, Whalley C, Poxon C, Wanigasooriya K, Pickles O, Aldera EL, Popakonstantinou D, Morley GL, Walker EM, Zielinska AE, McLoughlin D, Webster C, Plant T, Ellis A, Richter A, Kidd IM, Beggs AD (2020). Rapid implementation and validation of a cold-chain free SARS-CoV-2 diagnostic testing workflow to support surge capacity. J Clin Virol.

[8] Alcoba-Florez J, Gonzalez-Montelongo R, Inigo-Campos A, Gargia-Martinez de Artola D, Gil-Campesino H, Ciuffreda L, Valenzuela-Fernandez A, Flores C, The Microbiology Technical Support Team (2020). Fast SARS-CoV-2 detection byRT-qPCR in preheated nasopharyngeal swab samples. Int J Infect Dis.

[9] Kleiner M, Thorson E, Sharp CE (2017). Assessing species biomass contributions in microbial communities via metaproteomics. Nat Commun.

[10] Peschek N, Hoyos M, Herzog R, Förstner KU, Papenfort K (2019). A conserved RNA seed-pairing domain directs small RNA-mediated stress resistance in enterobacteria. EMBO J.

[11] Colosimo DA, Kohn JA, Luo PM (2019). Mapping interactions of microbial metabolites with human G-protein-coupled receptors. Cell Host Microbe.

[12] Ramalho J, Martins CSW, Galvão J (2019). Treatment of human immunodeficiency virus infection with tenofovir disoproxil fumarate-containing antiretrovirals maintains low bone formation rate, but increases osteoid volume on bone histomorphometry. J Bone Miner Res.

[13] Corman VM, Muth D, Niemeyer D, Drosten C (2018). Host and sources of endemic human coronaviruses. Adv Virus Res.

[14] Malik YA (2020). Properties of coronavirus and SARS-CoV-2. Malaysian J Pathol.

[15] Ziebuhr J, Herold J, Siddell SG (1995). Characterization of a human coronavirus (strain 229E) 3C-like proteinase activity. J Virol.

[16] Pan Y, Zhang D, Yang P, Poon LL, Wang Q (2020). Viral load of SARS-CoV-2 in clinical samples. Lancet Infect Dis.

[17] Piras A, Rizzo D, Longoni E, Turra N, Urru S, Saba PP, Musumano L, Bussu F. Nasopharyngeal swab collection in the suspicion of COVID-19. Am J Otolaryngol. 2020:102551. 10.1016/j.amjoto.2020.102551 Online ahead of print. 10.1016/j.amjoto.2020.102551 PMC7255165 32487335

[18] SoRelle JA, Frame I, Falcon A, et al. Clinical Validation of a SARS-CoV-2 Real-Time Reverse Transcription PCR Assay Targeting the Nucleocapsid Gene [published online ahead of print, 2020 Jun 1]. J Appl Lab Med. 2020;jfaa089. 10.1093/jalm/jfaa089. 10.1093/jalm/jfaa089 PMC7314039 32483586

[19] Mendoza EJ, Manguiat K, Wood H, Drebot M (2020). Two detailed plaque assay protocols for the quantification of infectious SARS-CoV-2. Curr Protoc Microbiol.

[20] Vosters A, Arbyn M, Baay M, Bosch X, Sanjose SD, Hanley S, Karafillakis E, Lupalco PL, Yarwood J, Damme PV (2017). Overcoming barriers in HPV vaccination and screening programs. Papillomavirus Res.

